# Genetic association stratified by HLA-DR3 and HLA-DR4 status reveals heterogeneity in pathways of progression to type 1 diabetes

**DOI:** 10.1007/s00125-026-06836-w

**Published:** 2026-08-31

**Authors:** Amber M. Luckett, Carolyn McGrail, Kathryn Murrall, Emily N. Griffin, Robin N. Beaumont, Gareth Hawkes, William A. Hagopian, Stephen S. Rich, Michael N. Weedon, Sarah Richardson, Richard A. Oram, Kyle J. Gaulton

**Affiliations:** 1 University of Exeter College of Medicine and Health, Exeter, UK; 2 Biomedical Sciences Graduate Program, University of California San Diego, La Jolla, CA USA; 3 Department of Pediatrics, University of California San Diego, La Jolla, CA USA; 4 Department of Pediatrics, Indiana University School of Medicine, Indianapolis, IN USA; 5 Center for Public Health Genomics, University of Virginia, Charlottesville, VA USA; 6 Royal Devon University Healthcare NHS Foundation Trust, Exeter, UK

**Keywords:** Autoantibodies, Genetic risk, Genome-wide association study, GWAS, HLA-DR3, HLA-DR4, Progression, Type 1 diabetes

## Abstract

**Aims/hypothesis:**

There are substantial differences in the clinical presentation of type 1 diabetes depending on the first-developing autoantibody, although the underlying mechanisms are poorly understood. The *DR3*-*DQ2* (DR3) and *DR4*-*DQ8* (DR4) haplotypes at the MHC locus associate with GADA and IAA as the first-developing autoantibodies, respectively, and can therefore be used as proxies for first autoantibody development in large cohort studies of type 1 diabetes cases and control individuals.

**Methods:**

We performed the first genome-wide association study of type 1 diabetes stratified by DR3 and DR4 status using 9091 type 1 diabetes cases and 14,157 control individuals from multiple cohorts. We estimated heritability and genetic correlation between type 1 diabetes with DR3 and DR4 (DR3-T1D and DR4-T1D, respectively), and with other immune and glycaemic phenotypes. We assessed heterogeneity in effects on type 1 diabetes between DR3 and DR4 individuals at known type 1 diabetes loci. We determined enrichment of type 1 diabetes heritability in DR3 and DR4 among variants in cell-type-specific *cis-*regulatory elements (cREs) and biological pathways, and annotated risk variants in cREs.

**Results:**

We observed only moderate genetic correlation between DR4-T1D and DR3-T1D (*r*_g_=0.6), which was lower compared with stratifications based on age of onset and sex, and distinct patterns of genetic correlations with other autoimmune diseases. Among type 1 diabetes-associated loci, the *IL2* locus had significantly larger effect on type 1 diabetes in DR4 while several other loci (*TAGAP*, *KLRG1*) had more nominal heterogeneity. There was stronger enrichment of DR4-T1D-associated variants in T cell cREs and T cell-related pathways, while DR3-T1D-associated variants were specifically enriched in mast cell cREs and secretion-related pathways. We finally prioritised specific loci annotated in mast cells with stronger effects on type 1 diabetes in DR3 individuals.

**Conclusions/interpretation:**

We performed the first GWAS of type 1 diabetes stratified by DR3 and DR4 status, which revealed heterogeneity in genetic risk and biological mechanisms dependent on high-risk HLA background.

**Graphical Abstract:**

**Figure:**
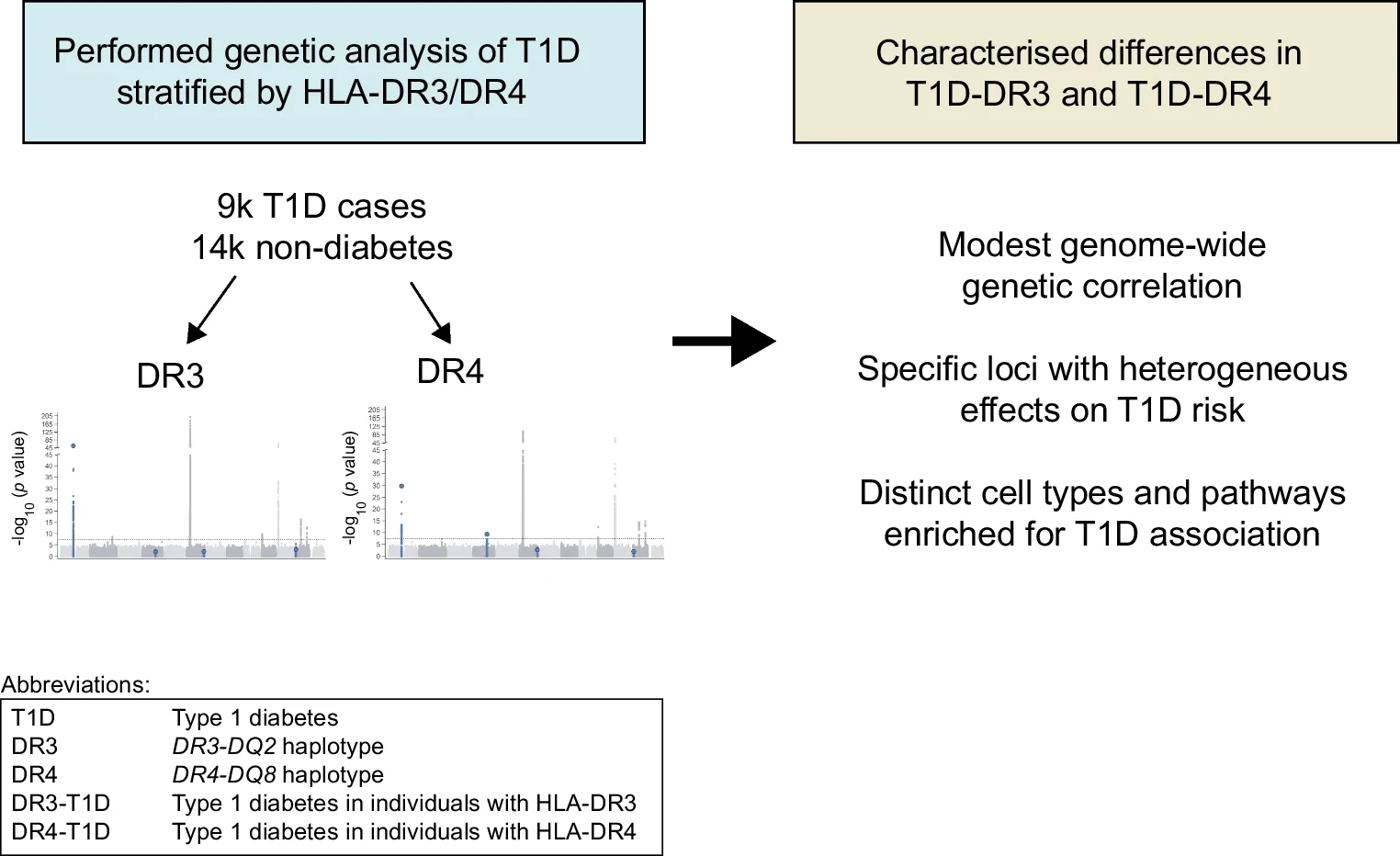

**Supplementary Information:**

The online version contains peer-reviewed but unedited supplementary material available at https://doi.org/10.1007/s00125-026-06836-w.

**Figure:**
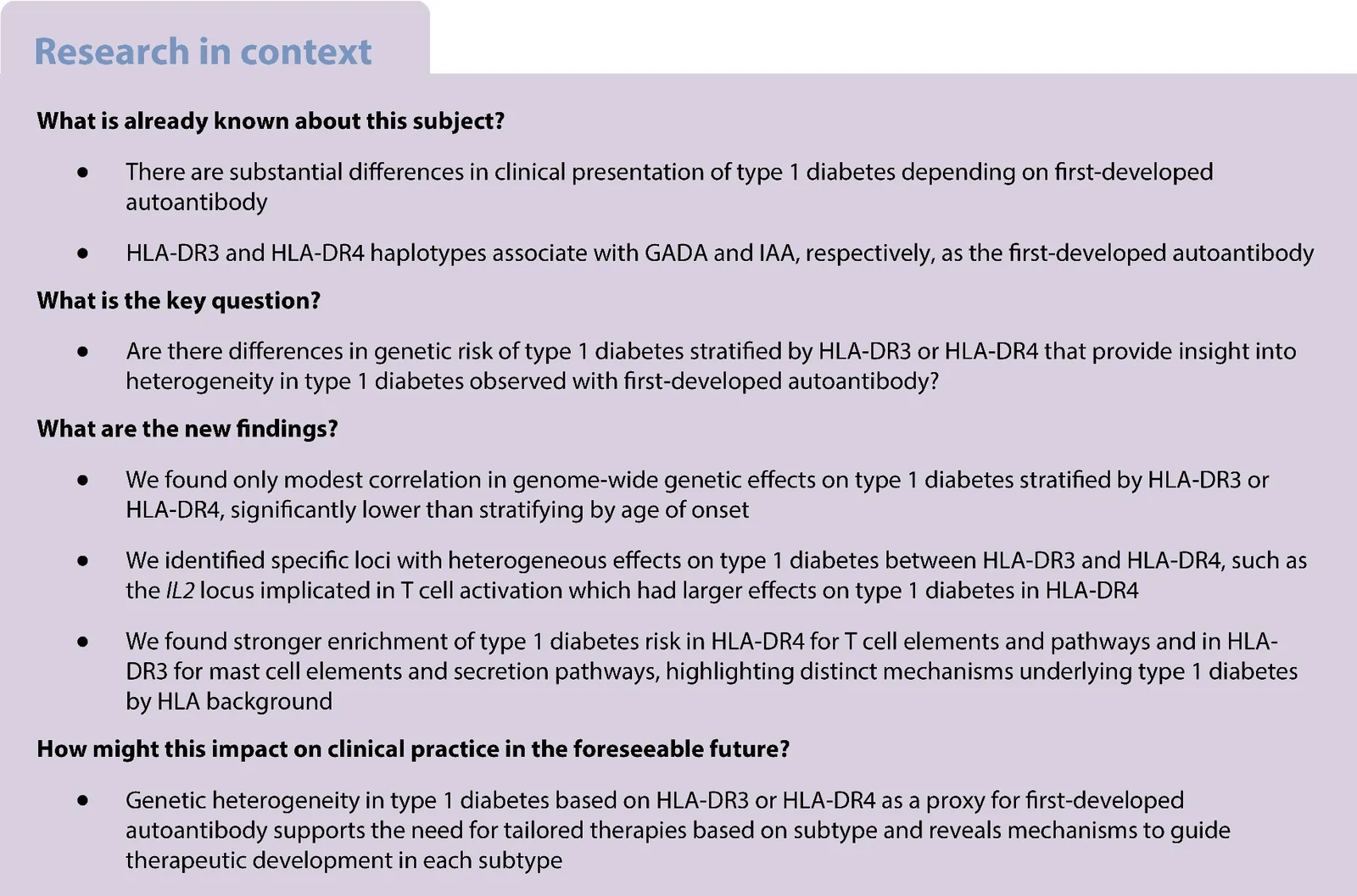

## Introduction

Type 1 diabetes is a complex disease characterised by autoimmune destruction of beta cells, but the underlying aetiology is not well understood [1]. The timing of progression to clinical diabetes varies widely across individuals, ranging from very early in childhood to adult onset [2, 3]. The development of autoantibodies (AAB) against islet proteins such as glutamic acid decarboxylase (GAD) and insulin (GADA and IAA, respectively) is a hallmark of disease progression in many type 1 diabetes cases. Recent studies have argued there are distinct endotypes of type 1 diabetes marked by the first-appearing AAB, where GADA-first type 1 diabetes typically has later onset compared with IAA-first type 1 diabetes [2, 3]. Insufficient data from cohort studies and the transient nature of AAB, however, have prohibited a deeper understanding of potential heterogeneity in disease processes leading to the development of GADA-first and IAA-first type 1 diabetes [2, 4].

The major histocompatibility complex (MHC) encodes human leukocyte antigen (HLA) genes vital in adaptive and innate immune responses [5], and the MHC locus confers substantial type 1 diabetes risk in European ancestry individuals. The largest type 1 diabetes risk factors at the MHC locus include the HLA haplotypes DRB1*03:01–DQA1*05:01–DQB1*02:01 (*DR3-DQ2*, denoted DR3) and DRB1*04:XX–DQA1*03:01–DQB1*03:02 (*DR4-DQ8*, denoted DR4) [5–8]. There is evidence to suggest a relationship between HLA haplotypes and the first-appearing AAB in individuals who develop type 1 diabetes, where DR3 associated with 3.5-fold increased rate of GADA-first AAB and later disease onset and DR4 with 2.4-fold increased rate of IAA-first AAB and earlier disease onset [2, 9, 10]. In addition, individuals with DR4 are more responsive to preventative type 1 diabetes treatments targeting T cell responses such as methyldopa and teplizumab, which supports the finding that distinct processes impact type 1 diabetes in different HLA backgrounds [4, 11, 12].

We hypothesised that identifying differences in genome-wide genetic risk of type 1 diabetes in individuals with DR3 or DR4 (DR3-T1D and DR4-T1D, respectively) would help reveal novel insights into differing processes involved in type 1 diabetes progression, including those related to first-developed AAB. Large type 1 diabetes case and control cohorts with genetic data enable the performing of genetic association studies of type 1 diabetes stratified by DR3 or DR4 as a proxy for first-developed AAB where cohort sizes are much smaller*.* We therefore performed a genome-wide association study of type 1 diabetes in individuals stratified by DR3 and DR4 and characterised heterogeneity in type 1 diabetes genetic risk and genomic mechanisms.

## Methods

### Study cohorts and genotype imputation

We compiled genotype data from 17,413 type 1 diabetes and 37,570 control individuals of European ancestry from available cohorts in dbGaP and EGA (ASP-GRID, DCCT, GENIE-UK, GoKIND, T1DGC, WTCCC, 1958 Birth cohort, eMERGE, HRS, NIMH) and from Exeter, UK (Extremely Early-Onset Type 1 Diabetes [EXE-T1D], EXTEND, TIGI, DARE, StartRight). Detailed cohort information and accession numbers are provided in electronic supplementary material (ESM) Table 1. European ancestry individuals were used in this analysis due to the higher frequency of DR3 and DR4. Individuals with the first four PCs >3 interquartile ranges from the 25th and 75th percentiles of PCs from the European 1000 Genomes Project [13] were considered non-European and excluded. Type 1 diabetes case cohorts were matched to control cohorts consistent with country of origin and genotype array, as previously described [14, 15]. Prior to imputation, we applied the HRC Imputation Preparation program (v4.2.9, https://www.well.ox.ac.uk/~wrayner/tools/) and used PLINK (v1.90, https://www.cog-genomics.org/plink/) [16] to perform quality control to remove variants with a difference in allele frequency >0.2 compared with the HRC r1.1 reference panel. We then removed variants with MAF<1%, with missing genotypes >5%, in violation of Hardy–Weinberg equilibrium (HWE) (HWE *p*<1×10^−5^ in control cohorts and HWE *p*<1×10^−10^ in case cohorts) and with allele ambiguity. We removed samples with missing genotypes >5%, sex mismatch based on reported phenotypes, non-European ancestry and cryptic relatedness through identity-by-descent (IBD>0.2).

**Fig. 1:**
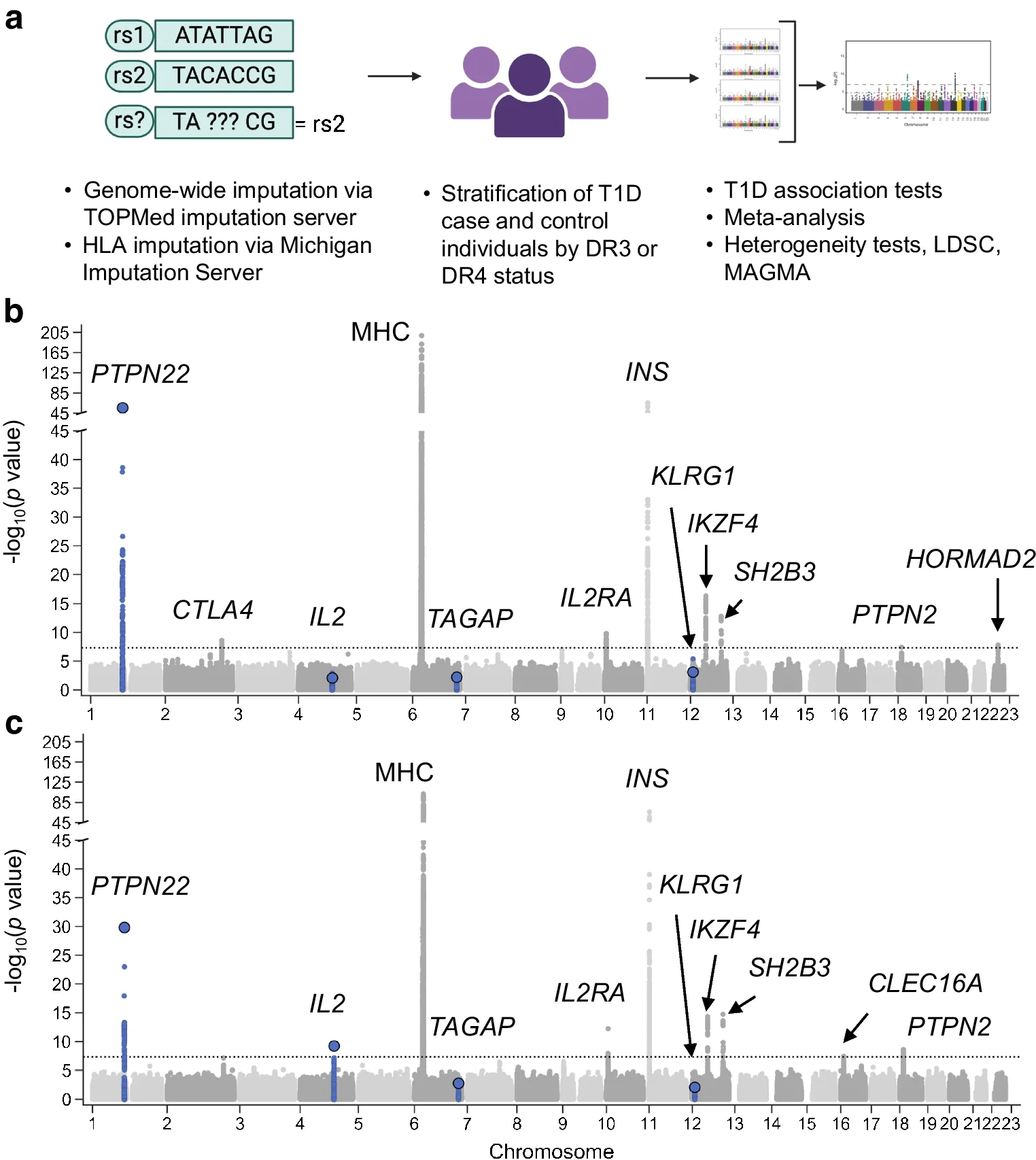
Genetic discovery for type 1 diabetes stratified by HLA-DR3 and HLA-DR4. (**a**) Overview of genetic association and heterogeneity tests for type 1 diabetes in DR3 and DR4 individuals. (**b**) Genome-wide type 1 diabetes association in individuals with DR3. log_10_ *p* values from meta-analysis of 4057 type 1 diabetes cases and 8043 non-type 1 diabetes control individuals. (**c**) Genome-wide type 1 diabetes association in individuals with DR4. log_10_ *p* values from meta-analysis of 5034 type 1 diabetes cases and 6114 non-type 1 diabetes control individuals. For both plots, significant loci are labelled with the nearest gene and loci with heterogeneous effects are coloured blue. Dotted line indicates genome-wide significance. T1D, type 1 diabetes

We used the TOPMed Imputation Server r2 panel and Michigan Imputation Server Multi-ethnic HLA reference panel for all samples (Fig. 1a) [17, 18]. For genome-wide imputation, we removed variants with an imputation accuracy score of *r*^2^<0.3. For HLA variant imputation, we used a threshold of *r*^2^<0.5 to remove variants. Variants that had a standard deviation in control allele frequency >0.055 across cohorts were also removed. Variants that passed quality control filters in all cohorts were tested for association.

We defined distinct DR3 and DR4 groups based on *DR* and *DQ* haplotype status using four-digit HLA alleles imputed from the Michigan Imputation Server. DR3 was identified by the presence of HLA DRB1*03:01–DQB1*02:01, while DR4 was identified by the presence of HLA DRB1*04:01/02/04/05/08–DQB1*03:02/04. We partitioned individuals by HLA *DR* and *DQ* haplotypes, resulting in 4057 DR3-T1D cases and 8043 DR3 control individuals and 5034 DR4-T1D cases and 6114 DR4 control individuals (ESM Table 1, Fig. 1a).

### Association testing and meta-analysis

For dbGaP cohorts, we tested TOPMed imputed variants for type 1 diabetes case and control association using Firth bias corrected logistic regression in EPACTS (v3.2.6; https://github.com/statgen/EPACTS) in both DR3 and DR4 groups. We tested variants with MAF>1% for association using sex and the first four genotype PCs as covariates. For Exeter cohorts, which have a smaller sample size, we used REGENIE (v3.2.6; https://rgcgithub.github.io/regenie/), which is suited to dealing with smaller cohorts by fitting a regression model to a subset of genetic markers prior to trait association testing using Firth bias corrected logistic regression [19]. We used variants with MAF>1% to generate LOCO (leave one chromosome out) predictions and used sex and the first four genotype PCs as covariates. We then performed association analysis in each cohort.

Summary statistics were combined across cohorts in a fixed effects inverse variance weighted meta-analysis. We removed HLA variants and calculated genomic inflation (λgc) for the DR3-T1D GWAS (λgc=1.02) and DR4-T1D GWAS (λgc=1.0). We considered variants significant at a *p* value threshold of 5×10^−8^, which represents Bonferroni correction for the estimated number of independent tests.

### Heritability and genetic correlation estimates

Using LD score regression (LDSC) we estimated the heritability of DR3-T1D and DR4-T1D, including and excluding the HLA region, using the liability scale [20]. We used a population prevalence of 0.0012 for DR3-T1D and DR4-T1D and a sample prevalence of 0.34 for DR3-T1D and 0.45 for DR4-T1D [21–23]. We then performed genetic correlation estimation between DR3-T1D and DR4-T1D, and within female participants with type 1 diabetes vs male participants with type 1 diabetes, and younger vs older onset type 1 diabetes (the lower median vs higher median type 1 diabetes age of onset from T1DGC, where the median was 8 years), as a positive control comparison due to high genetic correlation between these stratifications. We used a block jackknife approach [24] to estimate standard errors and to determine a difference between heritability estimates and genetic correlation estimates between HLA-stratified, sex-stratified and age-stratified type 1 diabetes, and then calculated the significance of these estimates using a *Z* test.

To determine genetic similarity between DR3-T1D, DR4-T1D and other traits, we used published GWAS summary statistics to estimate heritability, and genetic correlation estimates for 11 autoimmune traits (autoimmune thyroid disease, coeliac disease, Crohn’s disease, multiple sclerosis, primary biliary cirrhosis, primary sclerosing cholangitis, rheumatoid arthritis, systemic lupus erythematosus, type 1 diabetes, ulcerative colitis and vitiligo) and three non-autoimmune traits (type 2 diabetes, BMI and pancreas volume) [14, 25–35]. Sample and population prevalences were calculated from each GWAS and from current literature, respectively. We used Benjamini–Hochberg correction to determine whether correlations were significant (false discovery rate [FDR]<0.10).

### Type 1 diabetes variant heterogeneity testing between HLA-stratified groups

We tested heterogeneity in lead variants with MAF>1% from previously described type 1 diabetes fine-mapped signals [14] using a merged matrix of genotypes from dbGaP cohorts. We used PLINK to perform a Firth regression heterogeneity test (excluding MHC locus) to examine whether HLA status influenced the effect of established type 1 diabetes signals. For this interaction test, we included the covariates from logistic regression (PCs 1–4 and sex). We then performed a random-effects meta-analysis using the Genome-Wide Association Meta-Analysis (GWAMA) software (v2.2.2; https://genomics.ut.ee/en/tools) [36] to assess heterogeneity in effect size between DR3-T1D and DR4-T1D from all 87 type 1 diabetes variants.

### GWAS enrichment analysis

In the dbGaP cohorts, we used LDSC to perform a partitioned heritability analysis to estimate enrichment of type 1 diabetes heritability in cell-type-specific *cis*-regulatory elements (cREs) defined by CATlas [37]. We used the summary statistics for each group excluding the MHC locus and formatted the summary statistic file for input to LDSC. We computed cell-specific LD enrichment scores for DR3-T1D and DR4-T1D using the version 2.2 1000 Genomes baseline model and corrected for multiple tests (Benjamini–Hochberg correction) across all cell types using FDR. An FDR<0.10 was considered significant. We compared significant cell-specific LD enrichment scores for DR3-T1D and DR4-T1D using a block jackknife approach [24] where we calculated the coefficient difference across genomic blocks to estimate a standard error of the difference, and then calculated significance of these estimates with a *Z* test. We used MAGMA (v1.10; https://cncr.nl/research/magma/) with default parameters to perform gene set enrichment analysis in GO, KEGG and REACTOME pathways with DR3-T1D and DR4-T1D summary statistics. We assessed heterogeneity using a Cochran’s *Q* test. Pathways were considered significantly heterogenous at an FDR<0.10.

### Mast-cell-specific gene heterogeneity

From previously determined 99% credible sets for 136 type 1 diabetes signals, we identified known type 1 diabetes signals and determined overlaps within mast cell cREs defined by CATlas [37]. We then determined which variants were present in the meta-analyses for both DR3-T1D and DR4-T1D. We removed variants with a PIP<0.1 as these are less likely to be causal within the credible set. We then examined differences in strength of association to type 1 diabetes between the stratified HLA groups in credible sets that fell within accessible chromatin of mast cells.

### Ethics

Ethics approval for the DARE study was granted by the Devon & Torbay Research Ethics Committee (reference 2002/7/118). The StartRight study was approved by the Southwest–Cornwall and Plymouth NHS Research Ethics Committee (reference: 16/SW/0130). The EXE-T1D study has ethical approval from Derby Research Ethics Committee, Derby, UK (IRAS project ID 228082). The EXTEND/PRB study has ethical approval from Southwest–Cornwall & Plymouth NHS Research Ethics Committee, Bristol, UK (reference 14/SW/1089; 5 year extension following initial approval: reference 09/H0106/75). The TIGI study was approved by the Southwest–Central Bristol Research Ethics Committee (reference 13/SW/0312). The Institutional Review Board of the University of California San Diego gave ethical approval to analyse de-identified cohort data obtained from public sources such as dbGaP and EGA.

## Results

### Genome-wide association study of type 1 diabetes stratified by DR3/DR4 status

We obtained microarray genotype data of 17,413 type 1 diabetes case and 37,570 control European ancestry samples from 11 cohorts, and imputed genotypes into 308 million and 56,310 variants in the TOPMed v3 and Michigan HLA reference panels, respectively. We then stratified samples based on the presence of DR3 and DR4 haplotypes (see Methods), resulting in 12,100 DR3-T1D and control samples and 11,148 DR4-T1D and control samples. For these analyses we excluded individuals who were heterozygous DR3/DR4, as they could not be uniquely assigned to one group. We then tested variants with MAF>0.01 genome-wide for type 1 diabetes case and control association separately within the DR3 and DR4 groups (Fig. 1a).

In total, variants at 11 loci reached genome-wide significance (*p*<5×10^−8^) in either the DR3-T1D or DR4-T1D association analysis, all of which were loci previously reported in type 1 diabetes risk (Fig. 1b, c). Of these 11 loci, seven (*PTPN22*, *IL2RA*, *INS*, *IKZF4*, *SH2B3*, *PTPN2*, MHC) reached genome-wide significance in both analyses (Fig. 1b, c). Four additional loci reached genome-wide significance for type 1 diabetes in only one analysis, including *CTLA4* and *HORMAD2* in DR3 individuals and *CLEC16A* and *IL2* in DR4 individuals (Fig. 1b, c).

### Heritability and genetic correlation estimates

**Fig. 2:**
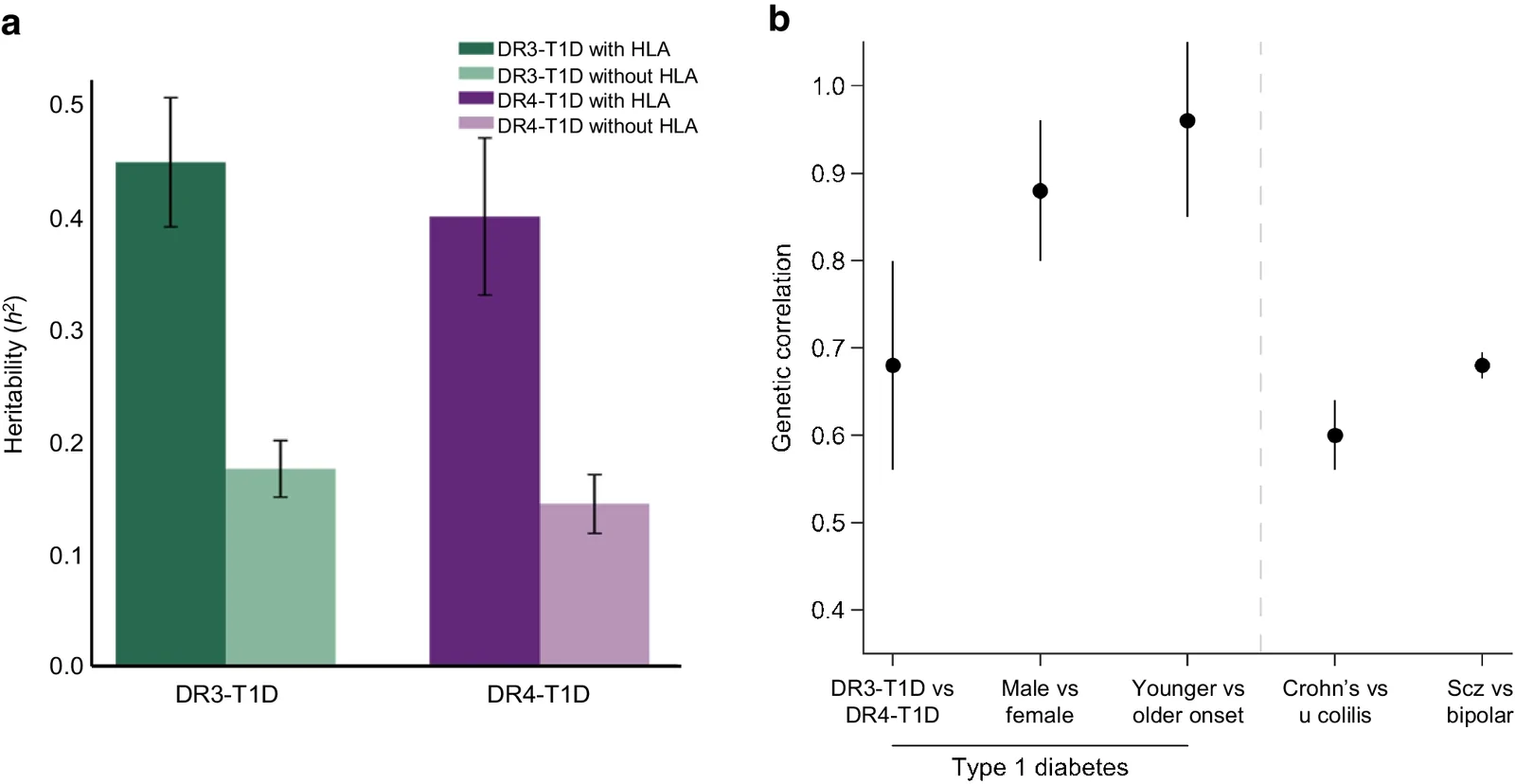
Heritability and genetic correlations in DR3 and DR4 type 1 diabetes. (**a**) Heritability estimates for DR3-T1D, including HLA variants (dark green) and excluding HLA variants (light green), and DR4-T1D including HLA variants (dark purple) and excluding HLA variants (light purple), with 95% confidence intervals as error bars. (**b**) Genetic correlation in type 1 diabetes association stratified by DR3 and DR4, median age of onset (diagnosis) and biological sex, with 95% confidence intervals as error bars. For comparison, the genetic correlations between schizophrenia and bipolar disorder (Scz vs bipolar) and between Crohn’s disease and ulcerative colitis (Crohn's vs u colitis) are also shown

We next calculated heritability estimates for DR3-T1D and DR4-T1D including and excluding the HLA region. The overall heritability for DR3-T1D (*h*^2^=0.45, SE=0.06) was higher than but not statistically different (*p*=0.51) from the DR4-T1D heritability (*h*^2^=0.40, SE=0.07) (Fig. 2a). After excluding the HLA region, both heritability estimates were as expected significantly reduced *(p*<0.05), with DR3-T1D having higher heritability (*h*^2^=0.17, SE=0.03) than DR4-T1D (*h*^2^=0.14, SE=0.03), although again not statistically significant (*p*=0.66) (Fig. 2a).

We next determined the correlation in genome-wide genetic effects on type 1 diabetes in DR3 and DR4 individuals. After excluding the HLA region, we found that DR3-T1D and DR4-T1D were moderately correlated (*r*_g_=0.68, SE=0.12) in genome-wide genetic effects. By comparison, we observed high correlation among type 1 diabetes associations between female and male participants (*r*_g_=0.88, SE=0.08), and between younger age of onset (<8 years based on T1DGC median) vs older age of onset type 1 diabetes (≥8 years based on T1DGC median) (*r*_g_=0.96, SE=0.11) (Fig. 2b). The correlation between DR3-T1D and DR4-T1D was significantly lower than the correlation between age of onset (*p*=9.86×10^−3^), but not sex (*p*=0.06), and was similar to the correlation between Crohn’s disease and ulcerative colitis and between schizophrenia and bipolar disorder. These results demonstrate that type 1 diabetes in DR3 and DR4 individuals shows less genetic similarity compared with previous stratifications of type 1 diabetes.

When assessing genetic correlation with other autoimmune and endocrine traits (excluding the HLA region), we found both shared and specific correlations between DR3-T1D or DR4-T1D and other traits (ESM Fig. 1a,b). After multiple test correction, both DR3-T1D and DR4-T1D were significantly (FDR<0.10) positively genetically correlated with rheumatoid arthritis (DR3-T1D *r*_g_=0.40, DR4-T1D *r*_g_=0.38), autoimmune thyroid disease (DR3-T1D *r*_g_=0.37, DR4-T1D *r*_g_=0.37) and systemic lupus erythematosus, although the correlation was stronger in DR4-T1D (DR3-T1D *r*_g_=0.19, DR4-T1D *r*_g_=0.41) (ESM Fig. 1b). Among traits with more specific enrichments, primary biliary cirrhosis was significantly (FDR<0.10) positively correlated with DR3-T1D only (DR3-T1D *r*_g_=0.22, DR4-T1D *r*_g_=0.12) and coeliac disease was significantly positively correlated with DR4-T1D only (DR4-T1D *r*_g_=0.3; DR3-T1D *r*_g_=0.05), as was vitiligo (DR4-T1D *r*_g_=0.31, DR3-T1D *r*_g_=0.16) (ESM Fig. 1b).

### Heterogeneity in variant effects on type 1 diabetes in DR3 and DR4 individuals

**Fig. 3:**
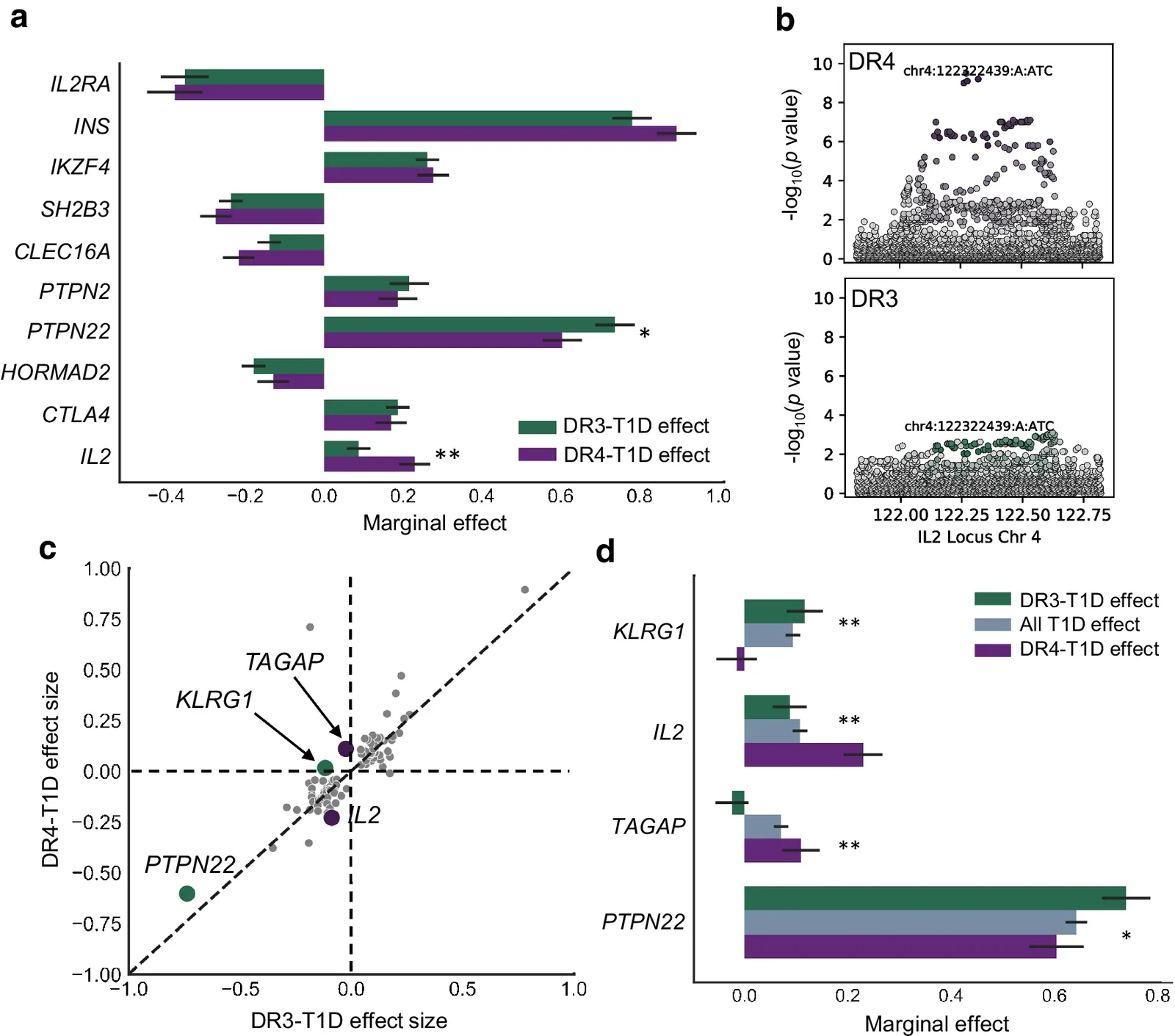
Genetic heterogeneity between type 1 diabetes in DR3 and DR4. (**a**) Plot of effect size of genome-wide significant loci in DR3-T1D (green) and DR4-T1D (purple). (**b**) Type 1 diabetes association at the *IL2* locus in DR4 (top) and DR3 (bottom) individuals. (**c**) Genetic effects on type 1 diabetes in DR3 and DR4 at 87 known type 1 diabetes loci. Larger points indicate signals with evidence for heterogeneity (*p*<0.05). Purple points indicate stronger effect size in DR4-T1D. Green points indicate stronger effect size in DR3-T1D. (**d**) Plot of effect size for heterogenous signals genome-wide between DR3-T1D (green) and DR4-T1D (purple) with overall type 1 diabetes effect size as a comparison. *p<0.05, **p<0.01. All error bars are standard errors

We next examined heterogeneity in the effects of variants at specific risk loci on type 1 diabetes risk between DR3-T1D and DR4-T1D. First, we determined whether the lead variants at the ten non-MHC loci with genome-wide significant association had heterogeneous effects on type 1 diabetes using an interaction test (see Methods). At nominal significance (*p*<0.05), the *PTPN22* locus had a stronger effect in DR3-T1D (DR3-T1D β=0.738, DR4-T1D β=0.604, *p*=0.03) while the *IL2* locus had a stronger effect in DR4-T1D (DR3-T1D β=0.0878, DR4-T1D β=0.230, *p*=0.006). After multiple test correction (FDR<0.10), only *IL2* had evidence for significant heterogeneity in effects between DR3-T1D and DR4-T1D (Fig. 3a, b).

Next, we more broadly determined whether lead variants across known type 1 diabetes risk loci outside of these ten significant loci had significant heterogeneity between DR3-T1D and DR4-T1D. We tested the heterogeneity of an additional 77 type 1 diabetes signals and found no additional loci with significant heterogeneity after FDR correction, although we found an additional two loci with nominal evidence (uncorrected *p*<0.05) (Fig. 3c–e). The *TAGAP* locus had a larger effect in DR4-T1D (DR3-T1D β=−0.024, DR4-T1D β=0.11, *p*=0.0077) and the *KLRG1* locus had a stronger effect in DR3-T1D (DR3-T1D β=0.11, DR4-T1D β=−0.015, *p*=0.0050). This heterogeneity is also reflected in stratified association results at these loci (ESM Fig. 2).

Finally, we sought to determine whether there was evidence for a broad difference in variant effects between DR3-T1D and DR4-T1D individuals. We used a random-effects meta-analysis to assess heterogeneity across the 87 lead type 1 diabetes risk variants. We observed nominal evidence of heterogeneity (*p*_*Q*_<0.05) at the *IL2*, *TAGAP*, *KLRG1* and *NOTCH2* loci, although these were not significant after multiple test correction (ESM Table 2).

### Cell-type-specific enrichment for type 1 diabetes association in DR3 and DR4

Given evidence for heterogeneity in genetic effects on type 1 diabetes between DR3-T1D and DR4-T1D individuals, we next sought to understand the biological mechanisms underlying these differences.

**Fig. 4:**
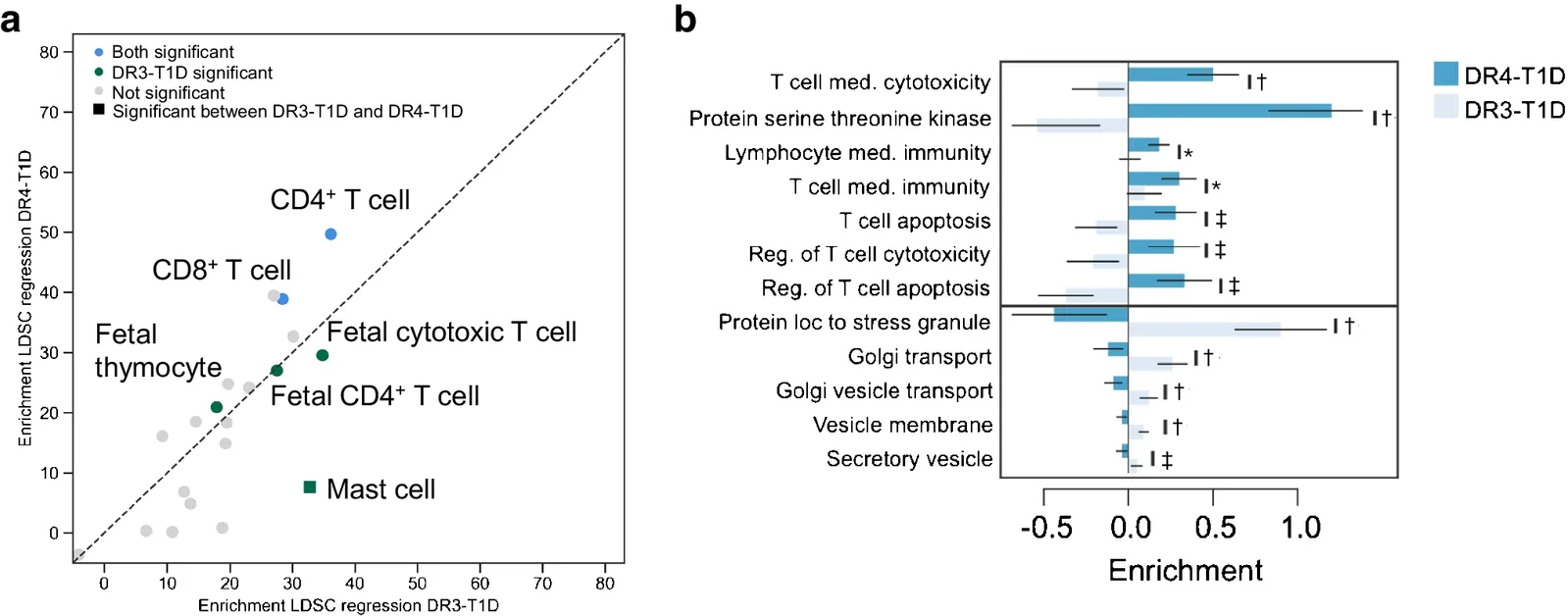
Functional annotations enriched in type 1 diabetes association in DR3 and DR4. (**a**) Enrichment scores of type 1 diabetes-associated variants in DR3 and DR4 for stimulated and unstimulated immune cell accessible chromatin sites using LDSC. Blue points are significant at FDR<0.10 in both DR3-T1D and DR4-T1D while green points are FDR significant only in DR3-T1D, and grey points are not significant. Square shape represents significant difference between DR3-T1D and DR4-T1D enrichment for mast cell cREs. (**b**) Enrichment of biological pathways for type 1 diabetes association in DR3 and DR4 using MAGMA. loc, localisation; med, mediated; reg, regulation. Darker blue bars are enrichment scores in DR4-T1D and light-blue bars are enrichment scores in DR3-T1D. ^†^FDR<0.10, ^‡^FDR<0.20, *p<0.05

We first performed enrichment analyses of DR3-T1D and DR4-T1D association for cREs active in immune cell populations using LDSC, and identified immune cell types with significant enrichment (FDR<0.10) in each group (ESM Table 3). In both the DR3-T1D and DR4-T1D groups, we observed significant enrichment (FDR<0.10) of type 1 diabetes-associated variants in CD4^+^ T cell and CD8^+^ T cell cREs (Fig. 4a, ESM Table 4). We also observed a strong enrichment of type 1 diabetes-associated variants in mast cell cREs for DR3-T1D that was significantly higher than in the DR4-T1D population (*p*=0.043).

We next identified biological pathways enriched for DR3-T1D- and DR4-T1D-associated variants using MAGMA. Among pathways enriched in either DR3-T1D or DR4-T1D, we further identified pathways with heterogeneity (FDR<0.10) in enrichment estimates between the two groups (Fig. 4b, ESM Table 5). Pathways with more pronounced enrichment in DR4-T1D included multiple related to T cell activity, signalling, apoptosis and immune responses. By comparison, pathways with stronger enrichment (FDR<0.10) in DR3-T1D were highly distinct and included those related to secretory vesicles, stress granules and several other processes. This reveals differences in biological pathways enriched for type 1 diabetes association in DR3 and DR4 individuals.

Type 1 diabetes-associated variants showed highly specific enrichment in mast cell cREs in DR3-T1D individuals and not DR4-T1D, suggesting mechanistic differences in cell types involved in disease progression, which we investigated further. We first identified type 1 diabetes-associated loci with candidate causal variants overlapping mast cell cREs. In total, seven type 1 diabetes loci contained at least one candidate variant (PIP>0.1) in a mast cell cRE, five of which had stronger effects in DR3-T1D compared with DR4-T1D (ESM Table 6). Among these were variants at the *FLI1* locus, where the *FLI1* gene has been implicated in histamine production in mast cells [38]. Next, we identified variants with sub-genome-wide significant type 1 diabetes association in mast cell cREs. Among loci at more nominal thresholds were several that had heterogeneous effects on type 1 diabetes between DR3-T1D and DR4-T1D and had no evidence for type 1 diabetes association in DR4. For example, variants at the 10q24 locus showed evidence for type 1 diabetes association in DR3 (β=−0.13, *p*=5.7×10^−5^) but not in DR4 (β=0.001, *p*=0.97) (ESM Fig. 3). These variants mapped in the *SUFU* gene, which has been implicated in regulating several key transcription factors in mast cells [39].

## Discussion

Genetic association of type 1 diabetes in individuals stratified by HLA-DR3 and HLA-DR4 haplotypes revealed evidence for heterogeneity in disease risk dependent on high-risk HLA background. The genetic correlation between DR3-T1D and DR4-T1D was significantly lower relative to other stratifications of type 1 diabetes such as age of onset. Furthermore, the correlation between DR3-T1D and DR4-T1D was comparable to correlations between different forms of other complex diseases such as psychiatric diseases (schizophrenia and bipolar disorder) [40] and inflammatory bowel diseases (Crohn’s disease and ulcerative colitis) [41]. In addition to broad heterogeneity in type 1 diabetes risk, we identified specific loci with heterogeneous effects between DR3 and DR4 including *IL2* which had more pronounced effects in DR4. Together this reveals that type 1 diabetes in a DR3 and DR4 background may represent heterogeneous sub-forms of type 1 diabetes at the genetic level, which is in line with the observed clinical differences in type 1 diabetes presentation dependent on first-appearing AAB.

We also identified distinct enrichments among type 1 diabetes-associated variants in DR3-T1D and DR4-T1D for regulatory elements in immune cell types, highlighting potential differences in underlying disease mechanisms. Mast cells were specifically enriched for type 1 diabetes-associated variants in DR3-T1D, which has not been described previously in type 1 diabetes GWAS, possibly due to a lack of DR4-T1D enrichment masking the effect. Mast cell activity may exacerbate type 1 diabetes progression in response to environmental exposures in DR3-T1D [42]. Altered mast cell activity in type 1 diabetes may thus have more temporal or spatial components, in addition to possible differences in gene expression [43, 44]. Our findings also provide further evidence of greater T cell response and enrichment in DR4-T1D. We also observed heterogeneity with increased effect in DR4-T1D at *IL2* which plays key roles in T cell activation and differentiation [45, 46]. The more pronounced role of T cells in DR4-T1D compared with DR3-T1D may guide personalised medicine using methyldopa, which binds to HLA-DQ8 to reduce T cell responses [12], and teplizumab, which binds to autoreactive T cells resulting in DR4 carriers responding better in absence of DR3 [11].

We utilised DR3 and DR4 status as a proxy for first-appearing antibody; however, there is only partial correlation between these HLA haplotypes and first-appearing AAB [2, 9, 10]. Future genetic studies of type 1 diabetes with first-appearing AAB will be needed to establish the extent of genetic heterogeneity driven by AAB status directly and how this relates to changes due to HLA-DR3/DR4 status. A current limitation, however, is the small available sample size of individuals where both first-developing AAB and genotyping data are available. In addition, the transient nature of type 1 diabetes AAB detection means prospective cohort studies such as TEDDY [22] and TrialNET [47], which monitor type 1 diabetes AAB progression, are ideally needed to establish robust definitions of AAB status for genetic analyses. Furthermore, additional type 1 diabetes risk factors including viral infection, dietary triggers and metabolic factors can affect AAB development [48], and probably interact with HLA background. Leveraging detailed environmental readouts together with HLA haplotype information will thus help more accurately predict first-developed AAB and can also potentially clarify how different HLA haplotypes interact with environmental risk factors to drive immune responses. Overall, given the link between HLA-DR3 and HLA-DR4 status and heterogeneity in disease risk in our study and considering the ability to readily profile genetic variants in individuals and the transient nature of AAB, HLA haplotype may represent a more robust marker of differences in type 1 diabetes progression.

Although we focused on genetic analysis of type 1 diabetes stratified by DR3 and DR4 status, there may be differences between the heterozygous combination of DR3 and DR4 compared with homozygous or single carriers of each haplotype. Heterozygous DR3/DR4 conveys the highest type 1 diabetes risk in European ancestry [7], and the association of heterozygous DR3/DR4 with first-developing AAB is unclear. However, the high type 1 diabetes risk conferred by this haplotype combination currently limits the number of control samples available for robust genetic association analyses in this group [2]. In addition, an estimated 10% of individuals with type 1 diabetes do not carry HLA-DR3 or HLA-DR4 haplotypes [15]. Recent analyses of non-DR3/DR4 individuals revealed differences in genetic risk of type 1 diabetes compared with DR3/DR4 individuals, where type 1 diabetes-associated variants showed reduced effect of T cell-related loci and stronger enrichment of inflammatory and beta cell-related pathways [15]. Given heterogeneity in genetic risk and disease mechanisms in non-DR3/DR4 type 1 diabetes, effective prevention of type 1 diabetes may require distinct therapies in this group as well.

The results of our study should have wide application to the design of future studies. First, DR3 and DR4 status could be considered in continued genetic association studies and risk prediction models of type 1 diabetes, for example, to understand risk variant interactions with HLA background [49]. Broader efforts to define heterogeneity in type 1 diabetes progression and pathogenesis and endotypes of type 1 diabetes should also consider the relationship to DR3 and DR4 status. Furthermore, given differences in the mechanisms of disease progression and likely therapeutic responses due to HLA background, future clinical trials may consider DR3 and DR4 status in their design. All these efforts could be extended to consider other high-risk HLA haplotypes, particularly in other populations where DR3 and DR4 are less common. Furthermore, there is considerable potential to expand this methodology to other autoimmune diseases, for example, stratifying rheumatoid arthritis by HLA-DRB1*04:01 and *04:04 [50, 51].

Overall, our results reveal broad differences in genetic risk of type 1 diabetes in individuals who carry HLA-DR3 or HLA-DR4 alleles, which supports recent evidence that type 1 diabetes is heterogeneous with multiple paths to disease. Furthermore, this argues for the need for tailored therapeutic approaches based on genetic background, for example, drugs targeting the innate immune response in DR3-T1D.

## Supplementary Information

Below is the link to the electronic supplementary material.

ESM (PDF 516 KB)

ESM Tables (XLSX 220 KB)

## Data Availability

Summary statistics from genome-wide association studies will be made available in the GWAS catalogue upon publication. Accession numbers for existing cohorts of type 1 diabetes case and control individuals obtained from dbGaP and EGA are listed in ESM Table 1. Exeter cohort clinical data can be used to identify individuals and are therefore available only through collaboration to experienced teams working on approved studies examining the mechanisms, cause, diagnosis and treatment of diabetes and other beta cell disorders. Requests for collaboration will be considered by a steering committee after an application to the Genetic Beta Cell Research Bank (https://www.diabetesgenes.org/current-research/genetic-beta-cell-research-bank/). Contact by email should be directed to RAO (R.Oram@exeter.ac.uk). All requests for access to data will be responded to within 14 days.

## Abbreviations

AAB: Autoantibodies
cRE: *cis*-Regulatory element
DR3: *DR3*-*DQ2* haplotype
DR4: *DR4*-*DQ8* haplotype
DR3-T1D: Type 1 diabetes in individuals with HLA-DR3
DR4-T1D: Type 1 diabetes in individuals with HLA-DR4
EXE-T1D: Extremely Early-Onset Type 1 Diabetes
FDR: False discovery rate
λgc: Genomic inflation
HLA: Human leukocyte antigen
HWE: Hardy–Weinberg equilibrium
LDSC: LD score regression
MHC: Major histocompatibility complex
