# Supplementary material for: Genetic association stratified by HLA-DR3 and HLA-DR4 status reveals heterogeneity in pathways of progression to type 1 diabetes
Source: Diabetologia. 2026 Aug 31;69(12):3580–91. doi: 10.1007/s00125-026-06836-w (PMC13714120; doi:10.1007/s00125-026-06836-w)
Supplement: Supplementary file 1 [file 125_2026_6836_MOESM1_ESM.pdf]

Electronic Supplementary Material

Table of contents

ESM Fig. 1
ESM Fig. 2
ESM Fig. 3
EXE-T1D consortium members
Additional acknowledgements

ESM Fig. 1

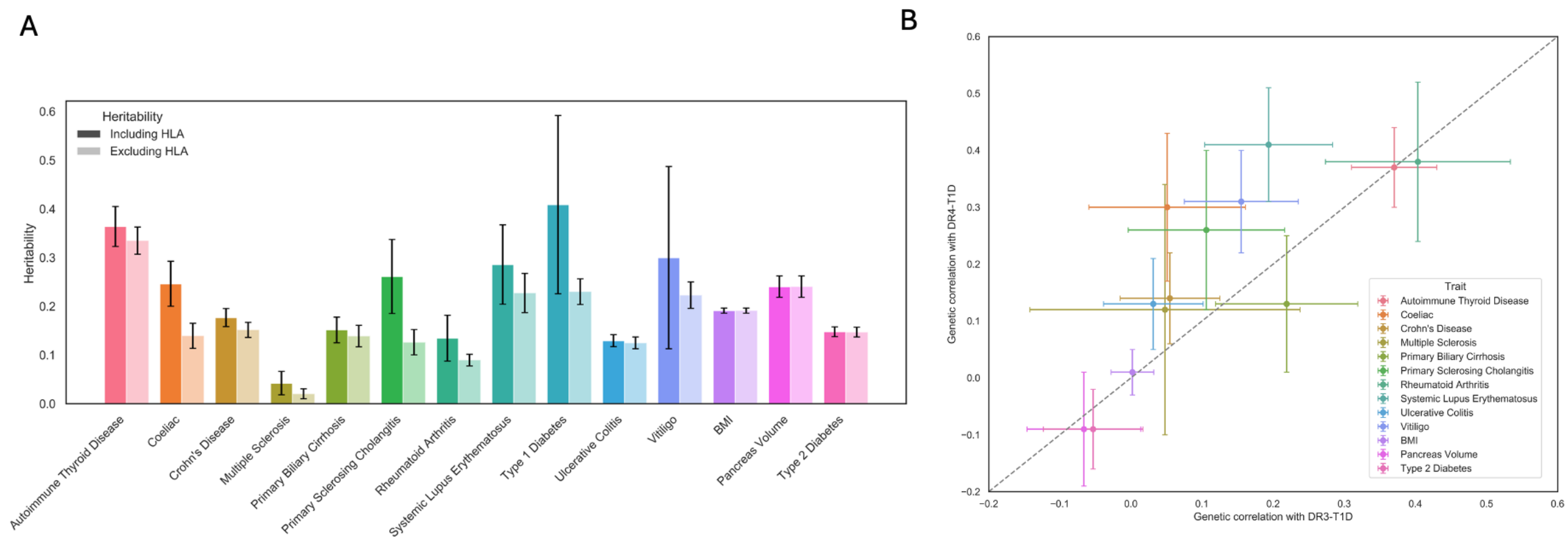

ESM Fig. 1. Heritability and Genetic Correlation with DR3-T1D and DR4-T1D. (A) Heritability estimates for autoimmune traits and additional polygenic traits. Darker colors represent estimate with HLA variants, lighter colors exclude HLA variants from estimates. Error bars are standard errors. (B) Genetic correlation of traits with DR3-T1D and DR4-T1D. Dashed line represents equal genetic correlation to DR3-T1D and DR4-T1D. Error bars are 95% confidence intervals.

ESM Fig. 2

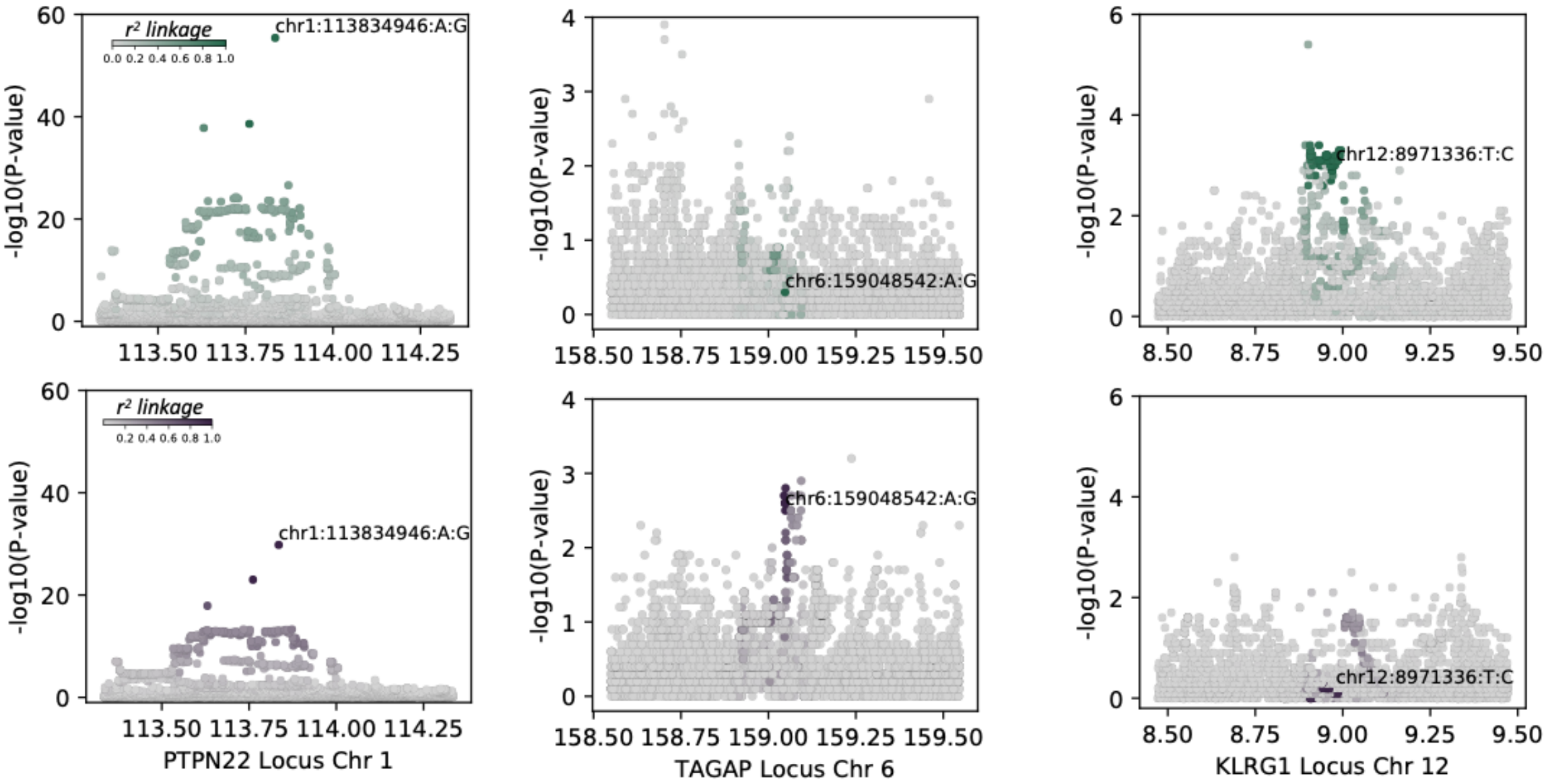

ESM Fig. 2. T1D signals with heterogeneity in DR3 and DR4. Locus plots for signals at *PTPN22* (left), *TAGAP* (middle) and *KLRG1* (right) with evidence for heterogeneity between DR3-T1D (green) and DR4-T1D (purple).

ESM Fig. 3

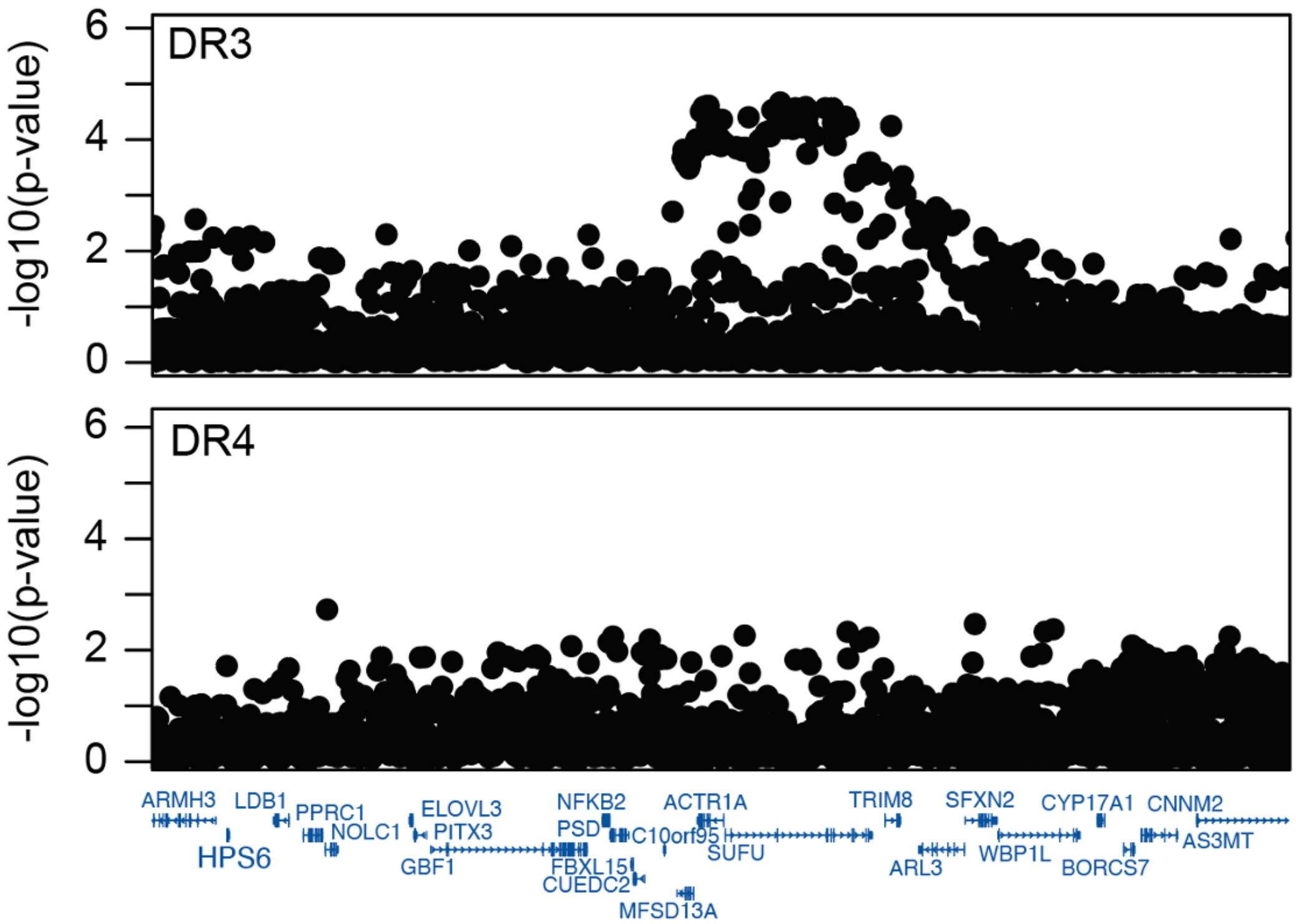

ESM Fig. 3. T1D association in DR3 and DR4 at the 10q24 locus. Locus plots showing T1D association of variants in DR3 (top) and DR4 (bottom) individuals at the 10q24 locus.

EXE-T1D Consortium members:

Department of Clinical & Biomedical Sciences, University of Exeter Medical School, Exeter, UK
Rebecca A Dobbs, Andrew T Hattersley, Michelle Hudson, Timothy J McDonald, Noel G Morgan, Matthew B Johnson, Georgia Bonfield, Suraj Ramchand, Dill Patel

Department of Immunobiology, School of Immunology & Microbial Sciences (SIMS), King's College London, UK
Timothy IM Tree, Paul Blair, Clara Domingo-Vila

Department of Internal Medicine, Cure One Type 1 Diabetes Center, Leiden University Medical Center, Leiden, the Netherlands
Bart O Roep

Center for Interventional Immunology, Benaroya Research Institute, Seattle, WA
Bradford Dimos, Cate Speake, Megan E Smithmyer

Translational Health Sciences, Bristol Medical School, University of Bristol, Southmead Hospital, Bristol, UK
Kathleen M Gillespie

Diabetes and Inflammation Laboratory, Centre for Human Genetics, Nuffield Department of Medicine, Bukhman Centre for Research Excellence in Type 1 Diabetes, Oxford NIHR Biomedical Research Centre, University of Oxford, Oxford, UK
Rachel E J Besser

Department of Paediatric Endocrinology and Diabetes, Oxford University Hospitals NHS Foundation Trust, Oxford, UK
Rachel E J Besser

Department of Paediatric Endocrinology and Diabetes, Birmingham Women's and Children's NHS Foundation Trust, Birmingham, UK
Renuka P Dias
Mendy Korer - Participant Parent Representative

Additional acknowledgements

The Diabetes Control and Complications Trial (DCCT) and its follow-up, the Epidemiology of Diabetes Interventions and Complications (EDIC) study, were conducted by the DCCT/EDIC Research Group and supported by National Institutes of Health (NIH) grants and contracts and by the General Clinical Research Center Program, National Center for Research Resources. The data from the DCCT/EDIC study were supplied by the National Institute of Diabetes and Digestive and Kidney Diseases (NIDDK) Central Repositories.

The Genetics of Nephropathy, an International Effort (GENIE), study was conducted by the GENIE Investigators and supported by NIDDK. The data from the GENIE study reported here were supplied by the GENIE Investigators from the Broad Institute of MIT and Harvard, Queens University Belfast, and the University of Dublin.

The Genetics of Kidneys in Diabetes (GoKinD) study was conducted by the GoKinD Investigators and supported by the JDRF, the Centers for Disease Control and Prevention, and the Special Statutory Funding Program for Type 1 Diabetes Research administered by NIDDK. The data (and samples) from the GoKinD study were supplied by the NIDDK Central Repositories. This manuscript was not prepared in collaboration with investigators of the GoKinD study and does not necessarily reflect the opinions or views of the GoKinD study, the NIDDK Central Repositories, or the NIDDK.

This research uses resources provided by the Type 1 Diabetes Genetics Consortium (T1DGC), a collaborative clinical study sponsored by the NIDDK, National Institute of Allergy and Infectious Diseases, National Human Genome Research Institute (NHGRI), National Institute of Child Health and Human Development, and JDRF and supported by grant U01 DK062418. The U.K. case series collection was additionally funded by JDRF and the Wellcome Trust and the National Institute for Health Research Cambridge Biomedical Centre at the Cambridge Institute for Medical Research, U.K., which is in receipt of Wellcome Trust Strategic Award 079895. The data from the T1DGC study were supplied by database of Genotypes and Phenotypes (dbGaP). This manuscript was not prepared in collaboration with investigators of the T1DGC study and does not necessarily reflect the opinions or views of the T1DGC study or the study sponsors.

This study makes use of data generated by the Wellcome Trust Case Control Consortium (WTCCC). A full list of the investigators who contributed to the generation of the data is available from https://www.wtccc.org.uk. Funding for the project was provided by the Wellcome Trust under award 076113.

Funding support for the Genome-Wide Association of Schizophrenia Study was provided by National Institute of Mental Health grants R01 MH67257, R01 MH59588, R01 MH59571, R01 MH59565, R01

MH59587, R01 MH60870, R01 MH59566, R01 MH59586, R01 MH61675, R01 MH60879, R01 MH81800, U01 MH46276, U01 MH46289 U01 MH46318, U01 MH79469, and U01 MH79470, and the genotyping of samples was provided through the Genetic Association Information Network. The data sets used for the analyses described in this manuscript were obtained from dbGaP (https://www.ncbi.nlm.nih.gov/gap) through dbGaP accession no. phs000021.v3.p2. Samples and associated phenotype data for the Genome-Wide Association of Schizophrenia Study were provided by the Molecular Genetics of Schizophrenia Collaboration (principal investigator Pablo V. Gejman, Evanston Northwestern Healthcare and Northwestern University).

Support for the collection of the data for Philadelphia Neurodevelopment Cohort was provided by grant RC2MH089983 awarded to Raquel Gur and RC2MH089924 awarded to Hakon Hakonarson. Subjects were recruited and genotyped through the Center for Applied Genomics (CAG) at The Children's Hospital in Philadelphia (CHOP). Phenotypic data collection occurred at the CAG/CHOP and at the Brain Behavior Laboratory, University of Pennsylvania.

Support for the Electronic Medical Records and Genomics (eMERGE) Network is as follows. The Group Health Cooperative/University of Washington receives funding support for the Alzheimer's Disease Patient Registry (ADPR), and the Adult Changes in Thought (ACT) study was provided by National Institute on Aging grant U01 AG006781 (Eric B. Larson, principal investigator). A gift from the 3M Corporation was used to expand the ACT cohort. DNA aliquots sufficient for genome-wide association studies from ADPR participants with probable Alzheimer disease who had been enrolled in Genetic Differences in Alzheimer's Cases and Controls (grant R01AG007584 to Walter Kukull, principal investigator) and obtained under that grant were made available to the eMERGE Network without charge. Funding support for genotyping, which was performed at Johns Hopkins University, was provided by NIH grant U01HG004438. Genome-wide association analyses were supported through NHGRI cooperative agreement U01HG004610 (Eric B. Larson, principal investigator). The Mayo Clinic provided samples and associated genotype and phenotype data used in this study. Funding support for the Mayo Clinic was provided through NHGRI cooperative agreement U0IHG004599 and by National Heart, Lung, and Blood Institute grant HL75794. Funding support for genotyping, which was performed at the Broad Institute, was provided by NIH grant U01HG004424. The Marshfield Clinic Research Foundation received funding support for the Personalized Medicine Research Project (PMRP) through NHGRI cooperative agreement U01HG004608, with additional funding from the National Institute for General Medical Sciences. The samples used for PMRP analyses were obtained with funding from Marshfield Clinic, Health Resources Service Administration Office of Rural Health Policy grant D1A RH00025, and Wisconsin Department of Commerce Technology Development Fund contract TDF FYO10718. Funding support for genotyping, which was performed at Johns Hopkins University, was provided by NIH grant U01HG004438. Northwestern University provided samples and data used in this study through the NUgene Project (https://www.cgm.northwestern.edu/cores/nugene/index.html). Funding support for the NUgene Project was provided by the Northwestern University's Center for Genetic Medicine, Northwestern University, and Northwestern Memorial Hospital. Assistance with

phenotype harmonization was provided by the eMERGE Coordinating Center (grant U01HG04603). This study was funded through the NIH NHGRI eMERGE Network (grant U01HG004609). Funding support for genotyping, which was performed at the Broad Institute, was provided by NIH grant U01HG004424.
